# Body Size Trade‐Offs Underpin Reproductive Success and Thermal Stress Tolerance in an Invasive Pest, the Small Hive Beetle

**DOI:** 10.1002/ece3.73629

**Published:** 2026-05-05

**Authors:** Bashiru Adams, Jian Chen, Esmaeil Amiri

**Affiliations:** ^1^ Delta Research and Extension Center Mississippi State University Stoneville Mississippi USA; ^2^ Biological Control of Pests Research Unit USDA‐ARS Stoneville Mississippi USA

**Keywords:** *Aethina tumida*, fitness trade‐offs, life history traits, parental effects, temperature

## Abstract

Body size is an important feature of organisms that correlates strongly with fitness, as it directly or indirectly influences nearly all biological phenomena. The body size of an organism is, in turn, shaped by many biological and physical factors that may not only directly affect the individual but also influence offspring through maternal investment or provisioning and transgenerational mechanisms. Body size differences have widely been observed in adult small hive beetles ( *Aethina tumida* Murray, SHBs), an invasive pest of honey bee colonies; however, little is known about the evolutionary and ecological implications of these variations. We hypothesized that parental body size influences reproductive performance, progeny fitness, and stress tolerance in SHBs. To test this, we paired different adult sizes and sexes of SHBs in rearing containers and compared their reproductive abilities and offspring fitness. We also exposed the progeny beetles to extreme temperatures and measured their thermal tolerance. A clear trade‐off emerged: larger beetles generated more offspring with lower fitness, while smaller adults produced fewer but higher‐fitness offspring. Additionally, larger SHB females showed greater tolerance to extreme temperatures, while small males were the most vulnerable. This study reveals that parental body size in SHB plays a pivotal role in shaping offspring reproductive traits and thermal stress tolerance. These findings highlight a potential mechanism by which SHB adapts and thrives across diverse and changing environments. Management strategies that exploit these life‐history trade‐offs could help shift populations toward weaker generations, thereby enhancing long‐term control effort.

## Graphical abstract

Body size differences have widely been observed in adult small hive beetles ( *Aethina tumida* Murray, SHBs), an invasive pest of honey bee colonies. We hypothesized that parental body size influences reproductive performance, progeny fitness, and stress tolerance in SHBs. We demonstrated that a clear trade‐off exists in SHBs: larger beetles produce more offspring with lower fitness, whereas smaller adults produce fewer but higher‐fitness offspring. Additionally, larger SHB females show greater tolerance to extreme temperatures, while smaller males are the most vulnerable.

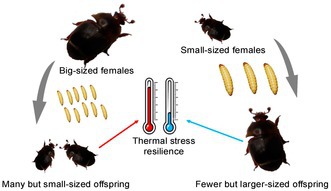

## 1 Introduction

Body size is a key factor in insect Ecology and Evolution because it is closely connected to survival, reproduction, and responses to environmental stressors (Amarillo‐Suárez et al. 2011; Kalinkat et al. 2015; Nufio et al. 2025). Variation in body size has been shown to affect fitness and life‐history traits (Amarillo‐Suárez et al. 2011; Clark et al. 2025; Kalinkat et al. 2015). This variation often results from trade‐offs among fitness components, such as offspring number versus offspring quality (Akhund‐Zade et al. 2021), or reproduction versus survival (Amiri et al. 2020; Leith et al. 2025). For instance, in the fruit fly *Drosophila melanogaster*, under space and resource limitation, offspring produced in large numbers are smaller and lighter, while those from smaller populations are larger and heavier (Akhund‐Zade et al. 2021). Similarly, in the tree hopper *Enchenopa binotata*, higher developmental temperatures enhance adult fertility, ensuring high juvenile survival and potentially preventing population declines due to global warming. However, pairing individuals reared at different developmental temperatures results in decreased morphological compatibilities between the sexes, reducing mating success and sperm transfer (Leith et al. 2025). These life‐history trade‐offs highlight the importance of body size variation from both ecological and evolutionary viewpoints.

Parental investment or provisioning, which in turn is affected by parental body size variation, affects offspring fitness in insects (Vasconcelos et al. 2025). The quantity and quality of offspring, along with their developmental time, survival rate, and stress resilience, are aspects of fitness that are affected by parental effects. For example, small adult females of the ectoparasitic wasp *Sclerodermus pupariae* produce larvae that take longer to reach the pupal stage than those from larger females (Gao et al. 2016). Additionally, in the seed beetle *Callosobruchus chinensis* , larger females lay bigger eggs, resulting in increased offspring survival and better performance when faced with challenging hosts (Yanagi and Tuda 2012). These examples indicate that parental investment, especially maternal provisioning, affects important biological processes linked to survival in insects. Parental investment represents an adaptation mechanism that often arises to enhance offspring survival and dispersion under stress conditions, especially thermal stress (Kohlmeier et al. 2024; Quan et al. 2024).

Temperature is a crucial environmental factor that affects the survival, development, and distribution of insects (Tonione et al. 2020; Nervo et al. 2021; Leith et al. 2025; Walzer et al. 2025). Thermal resilience, influenced by parental investment (Kohlmeier et al. 2024; Quan et al. 2024), may allow insects, particularly invasive species, to withstand seasonal changes, expand across diverse geographic regions, and survive climate change. Understanding these interactions offers valuable insights into insect invasion behavior.

Small hive beetle ( *Aethina tumida* Murray, SHB) is an important invasive pest of honey bees ( *Apis mellifera* ) (Neumann et al. 2016; Roth et al. 2022). Native to sub‐Saharan Africa, it has now spread to new environments, including North and South America, Australia, Europe, and Asia, causing significant ecological and economic damage to honey bee colonies (Neumann et al. 2016; Neumann and Elzen 2004). Management of SHB has relied heavily on chemical and mechanical/trapping controls over the past few decades; however, these methods have not been sustainable (Neumann et al. 2016; Bobadoye et al. 2025). More recent technologies against the beetle, such as RNAi, have shown promise but remain at the laboratory stage (Gu et al. 2025; Powell et al. 2017).

Like many insect species, there is a size difference between male and female SHBs (Cornelissen et al. 2024; Papach et al. 2019), a pattern known as female‐biased sexual size dimorphism. However, a wide range of body sizes can be seen within each SHB sex. Recent studies have demonstrated that the successful invasion behavior of SHBs hinges on their remarkable plasticity (Bai et al. 2022; Cornelissen et al. 2019; Liu et al. 2021; Noor‐Ul‐ane and Jung 2021). Traits such as high fecundity, rapid population growth, facultative feeding habits, and behavioral plasticity that allow SHBs to exploit host colony resources (such as honey bee hives) contribute to their invasion success (Neumann et al. 2016). Temperature has been shown to influence these life history traits and survival in SHBs (de Guzman and Frake 2007; Meikle and Patt 2011). However, the impact of other traits, such as body size, which may be influenced by maternal provisioning, on fitness and thermal‐stress resistance in SHBs remains poorly understood.

Here, we hypothesized that parental body size influences reproductive performance, progeny fitness, and thermal stress tolerance in SHBs. To test this hypothesis, we first investigated how variation in parental body size affects offspring numbers. We then assessed the influence of parental body size on offspring quality and overall fitness. Finally, we assessed the thermal tolerance of offspring of different sizes when exposed to extreme heat and cold stress. Together, these objectives aim to clarify the mechanisms that may enable the invasive SHB to persist across diverse environments and to inform management strategies that could shift populations toward weaker generations for improved long‐term control.

## 2 Materials and Methods

### 2.1 Small Hive Beetle Colony

**FIGURE 1:**
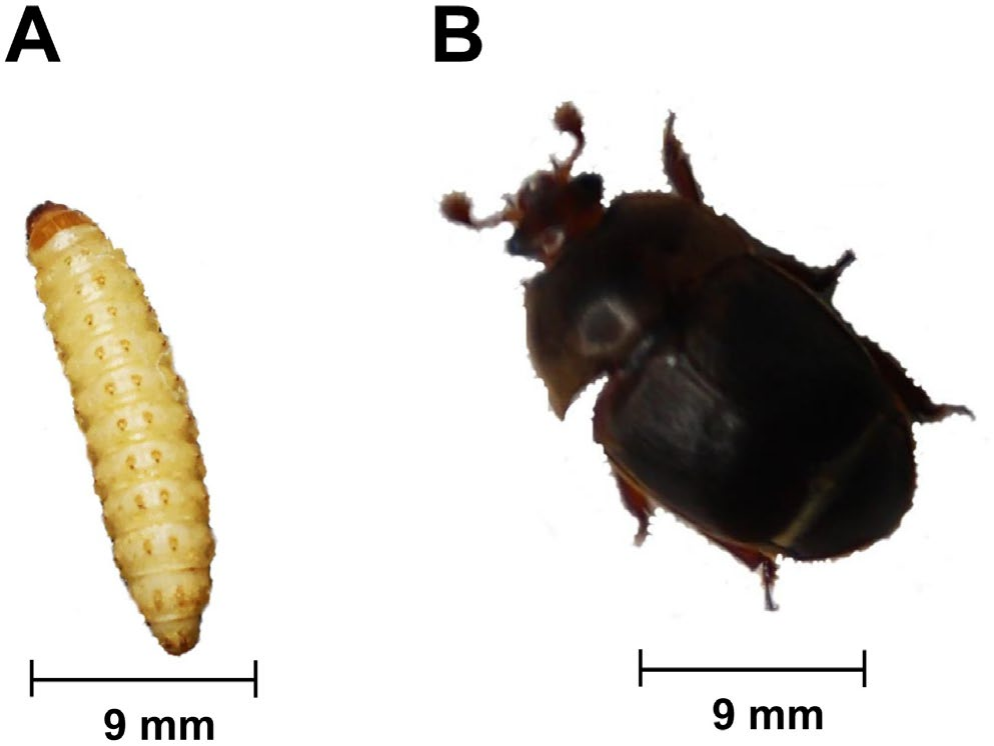
A picture of the small hive beetle ( *Aethina tumida* ) larva (A) and adult (B).

To initiate the experiment, live adult SHBs were collected from multiple apiaries near soybean, cotton, buckwheat, or corn farms and wild flowering plants across Mississippi, USA, during April–July 2025, and used to establish laboratory‐reared colonies. These beetles were considered the F0 generation and were used to produce offspring for subsequent experiments. They were reared in plastic containers (12 cm × 8 cm × 6 cm) at the Center for Pollinator Health laboratory at the Delta Research and Extension Center (DREC) in Stoneville, Mississippi. Pieces of combs (8 cm × 5 cm) containing pollen and honey were provided in the containers as regular food. To prevent laboratory‐induced differences in SHB individuals, beetle colonies were exposed to the same amount of food resources and environmental conditions. Four pairs of SHB adults were introduced into each container and kept in an incubator (30°C ± 2°C, 60%–70% RH, 12:12 L:D light/dark photoperiod) for mating and egg‐laying. The larvae that hatched (Figure 1A), fed for 2 weeks, and developed to the final larval stage (also referred to as the wandering stage) were transferred to sterilized sandy soil for pupation and adult development. Adult SHBs (Figure 1B) that had emerged (2–7 days old) were considered the F1 generation (parental generation) and used for the experiments.

### 2.2 Assessing the Life Traits of the SHB Groups

Upon emergence, each adult SHB was sexed as previously described (Papach et al. 2019; Roth et al. 2022). The body sizes, including the length (distance from the anterior part of the head to the posterior part of the abdomen) and the width (the widest distance from left to right or vice versa on the dorsal (top) part of the thorax), of randomly selected individuals (from experimental population) for each sex were then measured using a Leica S9i Digital Stereo‐microscope (Microscope Central, Feasterville‐Trevose, Pennsylvania, USA). Adults with body dimensions of length: 4.64 ± 0.14 mm and width: 3.34 ± 0.34 mm or higher were considered big‐sized, whereas those with length: 3.58 ± 0.15 mm and width: 2.09 ± 0.11 mm or less were considered small‐sized (Welch's *t*‐test, Length: *t*(35.404) = 10.298, *p* < 0.001 and Width: *t*(28.268) = 13.407, *p* < 0.001). Sexually matured SHBs of different body sizes were then paired as follows: (i) big female versus big male (BFBM), (ii) big female versus small male (BFSM), (iii) small female versus big male (SFBM), and (iv) small female versus small male (SFSM). For each experiment, one pair of males and females was placed in a plastic container (12 cm × 8 cm × 6 cm) containing a wax frame (8 cm × 4 cm × 3 cm) for egg laying. Each setup was replicated five times with different beetle pairs (10 insects per group). The beetles in each setup were fed 5 g of our own formulated pollen patty containing pollen (50%), honey (30%), glycerol (10%), and water (10%) weekly. Moist cotton wool was placed in each container to maintain a humid environment. The whole setup was kept in an incubator at 30°C ± 2°C and 60%–70% humidity. During the experiment, the number of larvae (F2 generation) that emerged was counted daily, and their body weight was measured individually on an analytical balance (Mettler Toledo ML104, Switzerland). Body length was also measured under a stereomicroscope, from the tip of the head to the posterior end of the body. Only ten larvae from each group were randomly selected and measured. Adult parental beetles (F1 generation) were maintained in containers until death, typically within 4–8 weeks.

In a second experiment, 10 SHB larvae (F2 generation) at their wandering stages (10–14 days old) of similar sizes originated from different F1 parental groups were placed in glass vials (20 mL, 28 mm outer diameter, FisherbrandTM Economic Glass EPA vials, Fisher Scientific, USA) containing 30 g of sterilized (oven‐heated at 65°C for 30 min to eliminate persistent soil‐borne pests and diseases) and moist soil to pupate until adults emerged. The vials were loosely capped to allow aeration. Each setup was replicated five times for each group (50 insects per group). The number of adults that emerged from each group was counted, and the weight of females and males were measured as described earlier. The development period (i.e., the number of days from larval emergence to first adult emergence) was then calculated. The adults that emerged were further grouped as big, small, or other (i.e., intermediate) based on body length and width, as previously described, for both males and females. The relationship between the SHB life traits was then assessed.

### 2.3 Assessing the Thermal Resilience of SHB Adult Groups

The responses of different adult SHB groups (F2 generation, 2–3 days old) to heat and cold stress were evaluated by exposing the insects to various temperature conditions. Four different SHB groups, including big females, small females, big males, and small males, were tested in this experiment. Big‐sized females and males (F2 generation) were offspring of small F1 females, whereas small females and males (F2 generation) were produced by big‐sized F1 female parents. Briefly, in this experiment, ten insects for each group were placed in glass vials (20 mL, 28 mm outer diameter, FisherbrandTM Economic Glass EPA vials, Fisher Scientific, USA), and 2 g of pollen patties (composition specified earlier) were provided as food (changed every two days). The vials were gently but loosely capped to allow airflow. The setup was replicated six times for each body size group (60 insects per group). Each setup was then placed in an incubator where temperature and humidity were controlled. A preliminary study was then conducted to test the beetles' response to conditions similar to those in our rearing chamber (30°C and 60%–70% RH). In the experiment measuring the beetles' response to heat stress, they were exposed to 35°C (simulating honey bee hive temperature) and two extreme temperatures (40°C and 45°C). For cold stress, they were exposed to 15°C, 10°C, and 5°C. In all the experiments, humidity ranged from 60% to 70%. The number of surviving insects was recorded daily for 10 days. Dead insects were removed from the setup every day.

### 2.4 Statistical Analysis

The count data from the SHB life traits, including the number of larvae and adults that emerged, the weight and length of larvae and adults, the development period, and the grouping of adults as big, small, or other, were analyzed using a generalized linear model (GLM) with a quasi‐Poisson distribution. The correlation among the life traits was assessed using Spearman's rank correlation. Survival data on SHB responses to thermal stress were analyzed using the Kaplan–Meier method (Kaplan and Meier 1958), where SHBs that survived to the end of the experiment were censored, and the potential impact of temperature was examined by comparing the survival curves of the lab rearing chamber group with those exposed to extreme temperatures during the 10‐day experimental period. Post hoc comparisons for the GLM were performed using Tukey's test, and the Kaplan–Meier survival curves were performed using the Wilcoxon test. In all analyses, comparisons were performed at a 5% significance level (*p* < 0.05). All data were analyzed using R software version 4.3.1 (R Core Team 2022) and the R Studio graphical user interface (version 2024.04.0) (Posit PBC 2024).

## 3 Results

### 3.1 Body Size Differentially Affects SHB Reproductive Potential and Offspring Fitness

**FIGURE 2:**
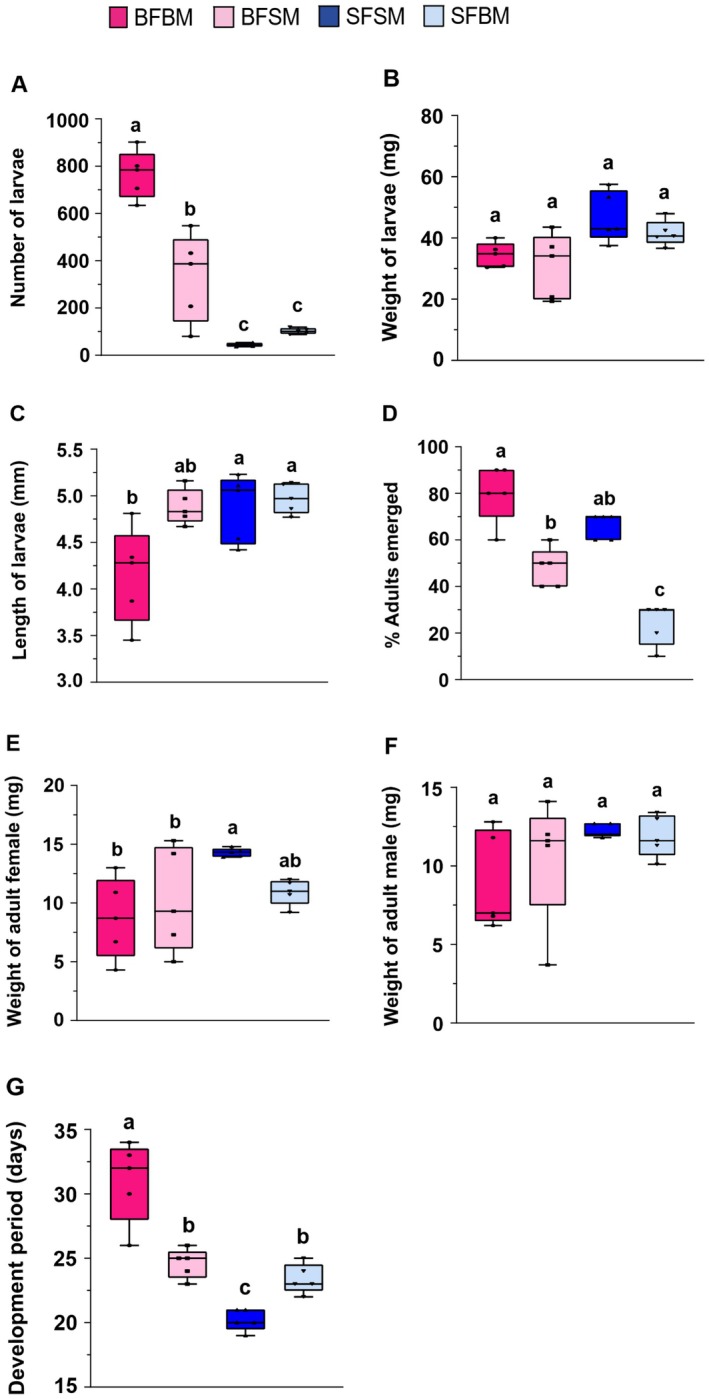
Effect of parental body size on life traits of small hive beetle offspring, (A) number of larvae produced, (B) larvae weight, (C) larvae length, (D) number of adults emerged, (E) female weight, (F) male weight, and (G) development period. (Generalized linear model: *p < 0.05, **p < 0.01, ***p < 0.001). Different lowercase letters on the bars indicate significant differences.

In the bioassay evaluating how body size influences parental reproductive potential, pairings of a BFBM produced the highest number of larvae (*χ* ^2^ = 115.6, df = 3, *p* < 0.001), followed by BFSM pairings (Figure 2A). The lowest larval numbers were recorded in SFSM and SFBM pairings (Figure 1A). Larval weight did not differ significantly among the groups (*χ* ^2^ = 41.323, df = 3, *p* = 0.348; Figure 2B). However, SFSM and SFBM pairings produced significantly longer larvae (*χ* ^2^ = 1.227, df = 3, *p* < 0.001), with lengths similar to those from the BFSM pairing. The shortest larvae were produced by the BFBM pairing (Figure 2C).

In the second experiment, which evaluated how body size influences offspring fitness, the BFBM pairing produced the highest number of emerging adults (*χ* ^2^ = 175.62, df = 3, *p* < 0.001), whereas the SFBM pairing produced the lowest (Figure 2D). Interestingly, for female offspring, SFSM and SFBM pairings yielded significantly heavier adults (*χ* ^2^ = 14.845, df = 3, *p* = 0.01), while BFBM and BFSM pairings produced the lightest females, with no difference between them (Figure 2E). In contrast, the weight of adult males did not differ significantly among the groups (*χ* ^2^ = 1.110, df = 3, *p* = 0.668; Figure 2F). Regarding the development time, offspring from pairs of BFBM required the longest time to develop (*χ* ^2^ = 12.143, df = 3, *p* < 0.001), whereas those from pairs of SFSM developed in the shortest time (Figure 2G).

**FIGURE 3:**
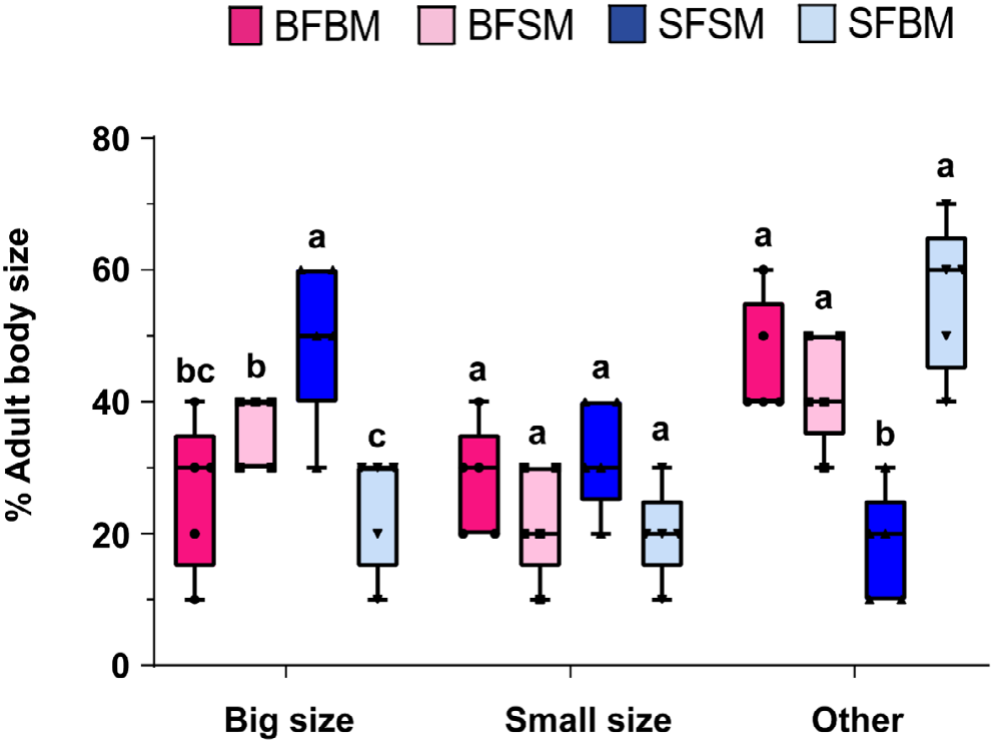
Effect of parental body size on the size of the first progeny in the small hive beetle. (Generalized linear model: *p < 0.05, **p < 0.01, ***p < 0.001). Different lowercase letters on the bars indicate significant differences.

Among the adults that emerged, pairs of SFSM produced the highest number of big‐sized adults (*χ* ^2^ = 60.066, df = 3, *p* < 0.001), whereas pairs of SFBM produced the fewest; however, this count did not differ in the offspring of the BFBM and BFSM (Figure 3). For small‐sized adults, no significant differences (*χ* ^2^ = 17.776, df = 3, *p* = 0.077) were detected among the groups (Figure 3). For adults of intermediate size, pairs of SFSM produced the fewest individuals (*χ* ^2^ = 109.35, df = 3, *p* < 0.001), while the BFBM, BFSM, and SFBM produced the highest numbers, with no differences among them (Figure 3). Across all experiments, the sex ratio ranged from 2:1 to 3:1, favoring females over males.

### 3.2 Parental Body Size Correlates With Offspring Fitness

**FIGURE 4:**
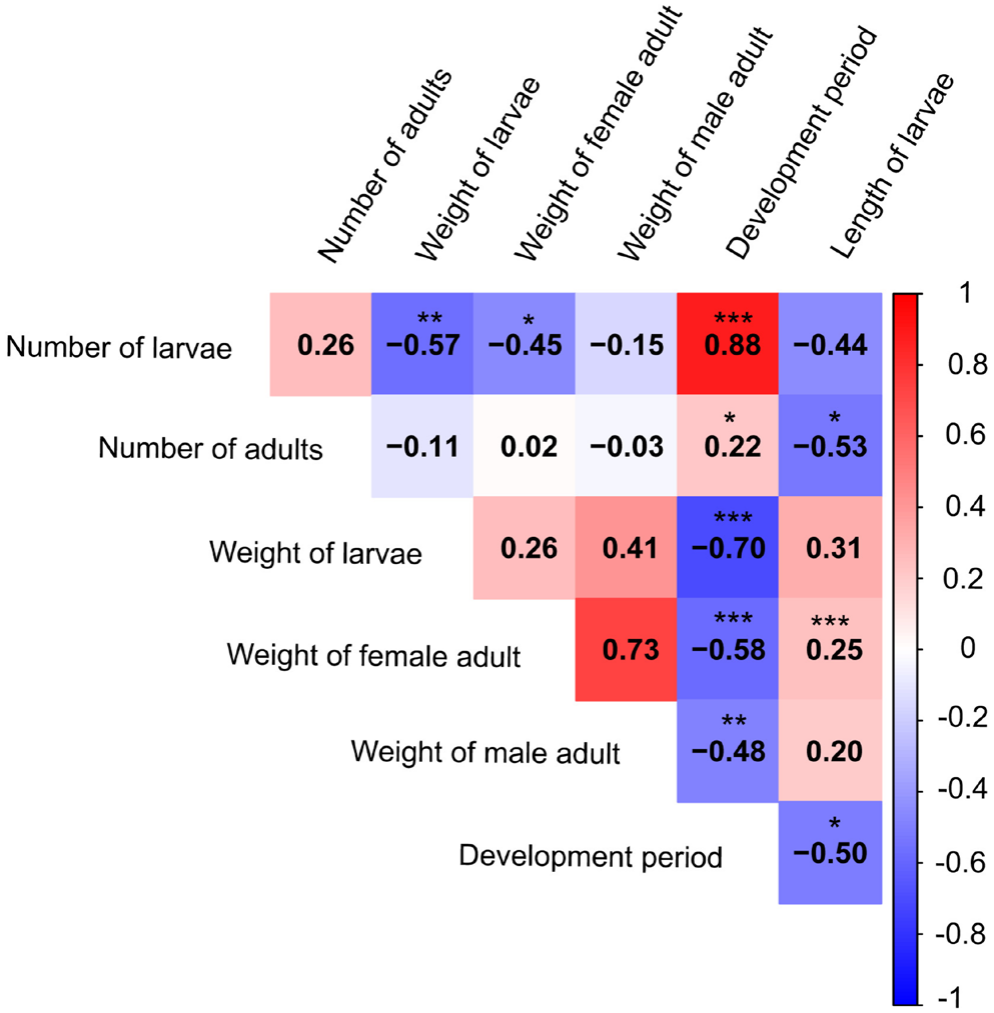
Correlograms illustrate the relationships between small hive beetle life traits. The numbers in each box represent the Spearman correlation coefficients (ρ) for each pair of life traits compared. The absolute value of the correlation coefficient (ρ) shows strength as weak (|ρ| < 0.3), moderate (|ρ| = 0.3–0.7), or strong (|ρ| > 0.7). Asterisks denote significant correlations (*p < 0.05, **p < 0.01, ***p < 0.001).

To investigate correlations among SHB life traits, a Spearman correlation was performed, and correlograms were constructed for each trait (Figure 4). A strong, positive, and significant correlation (*ρ* = 0.88, *p* < 0.001) was found between the number of larvae produced and the development time (Figure 4). A weak, positive, and significant correlation (*ρ* = 0.25, *p* < 0.001) was observed between larval length and adult female weight (Figure 4). A similar correlation was observed between the number of adults that emerged and the development period (*ρ* = 0.22, *p* < 0.05; Figure 4). In contrast, a significant negative correlation was observed between the number of larvae produced, their weight (*ρ* = −0.57, *p* < 0.01), and that of adult females (*ρ* = −0.45, *p* < 0.05; Figure 4). Additionally, a significant negative correlation was found between the development period and larval weight (*ρ* = −0.70, *p* < 0.001) and length of larvae (*ρ* = −0.50, *p* < 0.05), as well as the weight of both adult males (*ρ* = −0.48, *p* < 0.01) and females (*ρ* = −0.58, *p* < 0.001; Figure 4).

### 3.3 Body Size Affects SHB Tolerance to Thermal Stress

**FIGURE 5:**
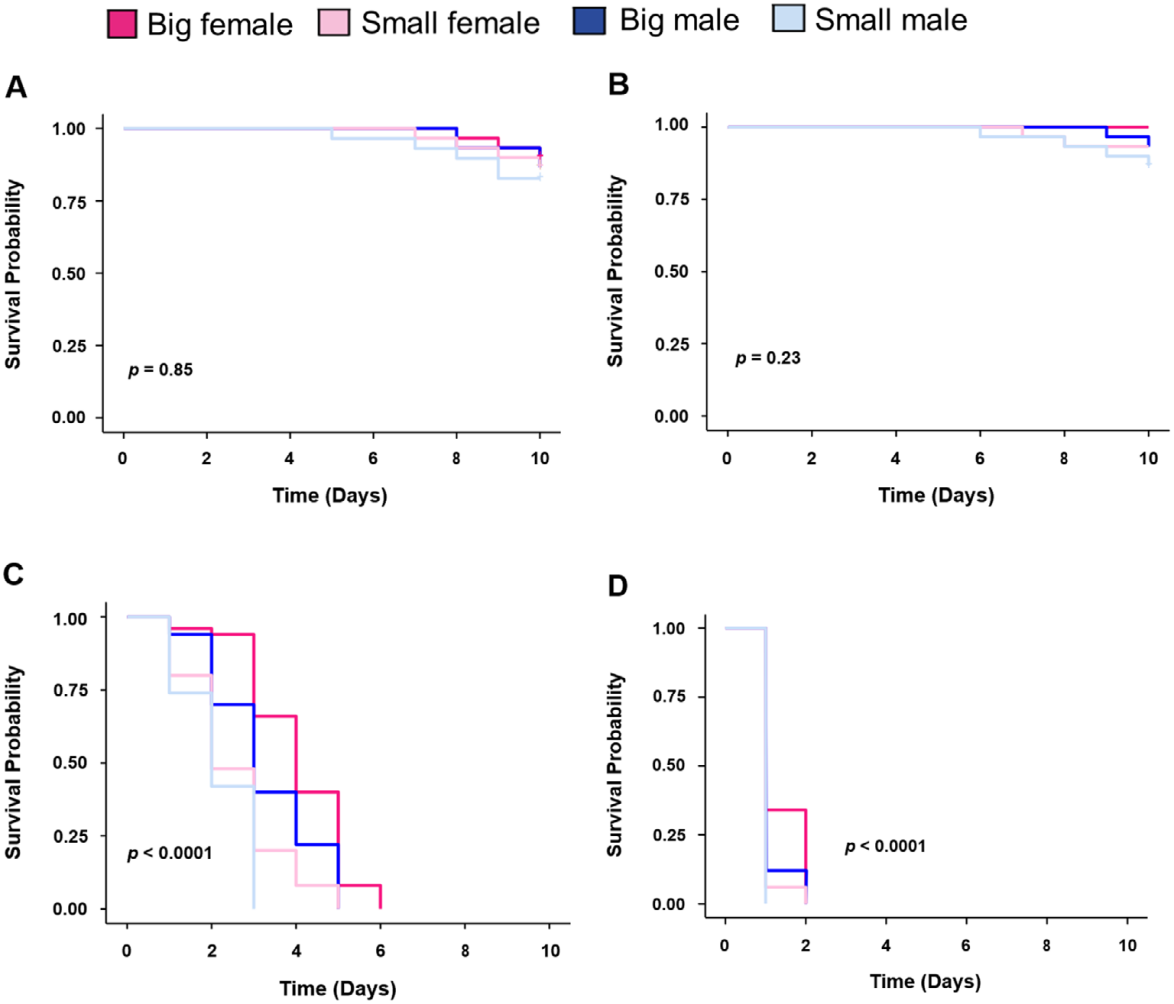
Heat stress exposure differentially impacts the survival of small hive beetles with varying body sizes (*n* = 6 replicates per group, i.e., 60 individuals), (A) preliminary experiment at 30°C, (B) temperature similar to inside a honey bee hive at 35°C, and two extreme temperatures at (C) 40°C and (D) 45°C. (Kaplan–Meier survival curves using the Wilcoxon test: *p < 0.05, **p < 0.01, ***p < 0.001).

The different SHB groups showed variable tolerance to cold and heat stress. In our preliminary study, exposing SHBs to 30°C, a temperature similar to that of our rearing chamber, did not significantly impact survival among the groups (*p* = 0.85; Figure 5A). Similarly, at 35°C, a temperature similar to that inside a honey bee hive, the beetles showed no significant difference in survival among the groups (*p* = 0.23; Figure 5B). However, at higher temperatures, big‐sized females exhibited the greatest resilience to heat stress at 40°C (*p* < 0.001; Figure 5C) and 45°C (*p* < 0.001; Figure 5D). Small‐sized males were the most vulnerable to heat stress. The beetles' responses followed a temperature‐dependent pattern, with increasing temperatures significantly reducing their survival.

**FIGURE 6:**
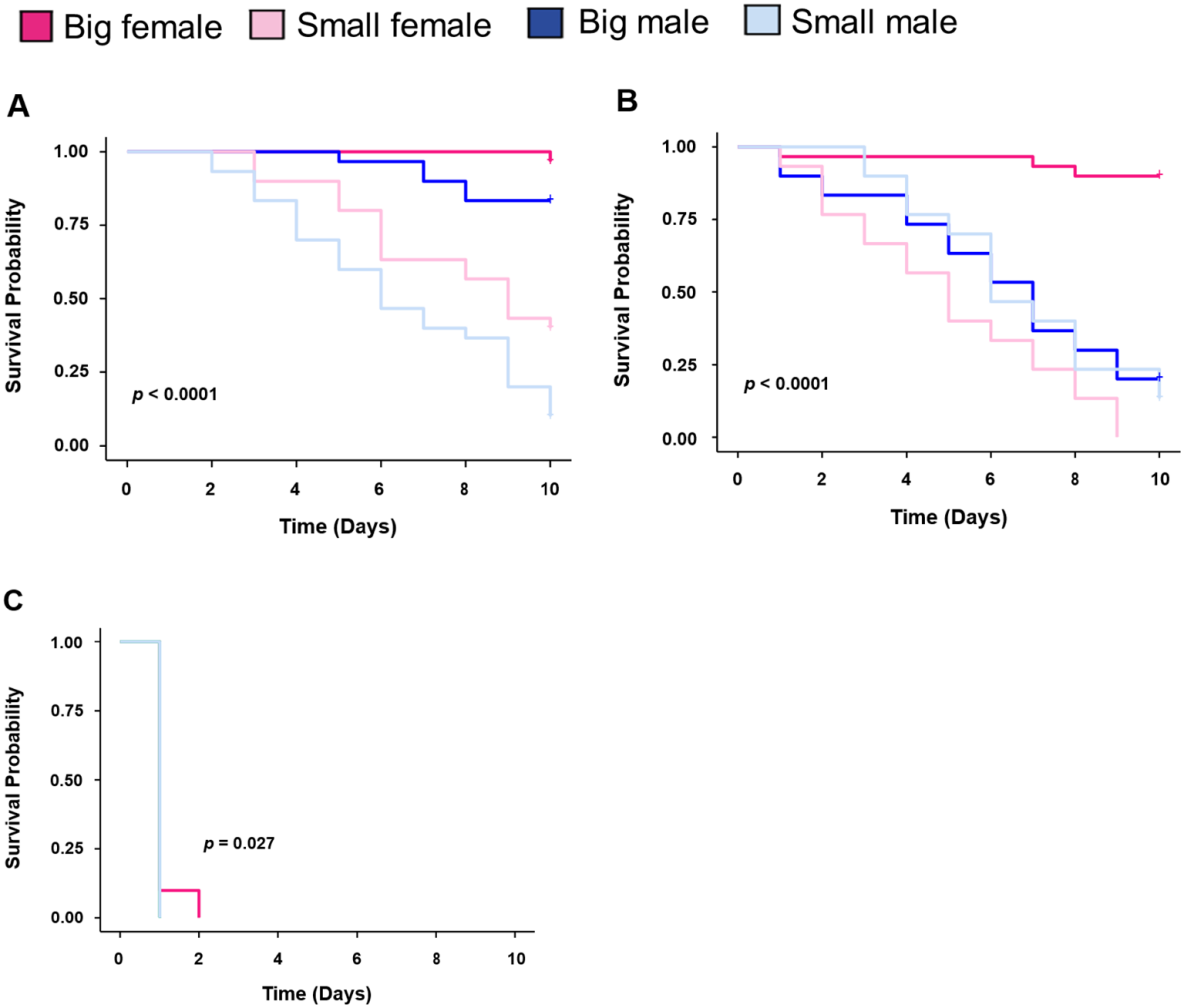
Cold stress exposure differentially impacts the survival of small hive beetles with varying body sizes (*n* = 6 replicates per group, i.e., 60 individuals), (A) 15°C, (B) 10°C, and (C) 5°C. (Kaplan–Meier survival curves using the Wilcoxon test: *p < 0.05, **p < 0.01, ***p < 0.001).

In response to cold stress, big‐sized females showed the greatest resilience at all tested temperatures, 15°C (*p* < 0.001; Figure 6A), 10°C (*p* < 0.001; Figure 6B), and 5°C (*p* = 0.027; Figure 6C), while small‐sized males were the most vulnerable. SHB's survival decreased as temperatures dropped. At the coldest temperature (5°C), the beetles barely survived for up to 2 days.

## 4 Discussion

This study demonstrates the ecological and evolutionary role of body size variation in the invasive SHB. We found that big‐sized females, regardless of the size of the male they mate with, produce more offspring than smaller females. Additionally, larger females produce offspring with a higher adult emergence rate, but they take longer to develop into adults. In contrast, smaller females, regardless of the size of the male they are paired with, produce fewer offspring that are bigger, longer, and heavier. Moreover, small‐sized females produce larger adults with a shorter development time. We also found a significant correlation between parental body size and their offspring's fitness traits, including larval number and length, adult number and weight, and development time. Irrespective of the type of thermal stress (heat or cold), big‐sized females were the most resilient, while smaller males were the most vulnerable. These findings reflect a classic “bet‐hedging” strategy. Bet‐hedging is an evolutionary tactic in which a species spreads its risks by producing different types of offspring or employing multiple strategies, ensuring that at least some individuals survive under unpredictable or changing conditions (Hopper 2018; Rowiński et al. 2021).

The differences in the number, size, length, and weight of offspring produced by big‐sized and small‐sized females suggest a link between maternal size and offspring fitness, which may reflect a maternal investment (nutrient allocation in eggs and hormone transfer) or a trade‐off between survival and reproduction. In beetles, larger females usually show higher fecundity and invest more resources per offspring than smaller conspecifics, as demonstrated in ground beetles (Knapp and Uhnavá 2014), seed beetles (Yanagi and Tuda 2012), and burying beetles (Clark et al. 2025). Nutritional status and hormonal signals such as juvenile hormone and ecdysteroids work together to regulate vitellogenesis and oogenesis (Knapp and Uhnavá 2014). This positive relationship between fecundity and size aligns with larger females having more stored nutrients and endocrine capacity to support oocyte development and offspring production (Chown and Gaston 2010; Knapp and Uhnavá 2014; Leyria et al. 2025). Moreover, big‐sized females have greater abdominal volume, enabling them to support larger ovaries and more ovarioles, potentially leading to greater egg mass production than in smaller females (Sarikaya et al. 2019; Tian et al. 2023). This could explain the observed variation in the number of offspring produced by SHB females of different body sizes in our study. This finding further supports previous studies showing that larger females produce more offspring than smaller ones in other beetle species (Kajita and Evans 2010; Steiger 2013). Smaller females may invest more resources per offspring, thereby improving progeny quality, but this comes at the cost of fewer offspring (Kindsvater and Alonzo 2014; Vijendravarma et al. 2010; Walker et al. 2008). This may explain the few, larger, longer, and heavier offspring produced by smaller SHB females in our study, suggesting a trade‐off between offspring number and offspring quality or a result of maternal investment. Small‐sized SHBs have fewer offspring, but their offspring develop faster and are larger, which may be related to their adaptation to harsh environments. Reducing development time helps vulnerable larvae quickly overcome a dangerous period, but previous studies have shown that under conditions of abundant nutrition (consuming high‐protein bee pupae), the larval stage can also be significantly shortened (Yang et al. 2024). This suggests that maternal investment in SHBs may combine with other external factors to improve offspring quality, which in turn influences offspring development time. It makes a lot of sense because advancing quickly through vulnerable juvenile stages can lower the overall risk of attack by honey bees or stress, making rapid development beneficial in harsh environments, commonly encountered by invasive species, including SHBs.

Interestingly, our results further show that, regardless of male size, the fitness of offspring from big‐sized SHB females followed the same pattern. This agrees with previous studies showing that paternal effects on offspring fitness are weaker, less consistent, or absent compared to maternal effects (Sahm et al. 2023; Vainikka et al. 2006). In a related beetle species, *Nicrophorus vespilloides* , no significant effect of male size on offspring body size across generations has been reported (Sahm et al. 2023). Similarly, in the mealworm beetle *Tenebrio molitor* , the survival and body size of offspring from males with more attractive pheromones do not differ from those of males with less attractive pheromones (Vainikka et al. 2006). Similar findings have been previously reported in other insect species (Bertram et al. 2016; Vijendravarma et al. 2010). For example, in *D. melanogaster* , the impact of parental diet on the developmental timing and weight of offspring fed poor‐quality food is influenced by maternal effects (Vijendravarma et al. 2010). Likewise, in the Jamaican field cricket, *Gryllus assimilis* , although females prefer larger males, the number and viability of offspring produced are not different from those produced when mated with non‐preferred smaller males (Bertram et al. 2016). Thus, these examples, combined with our results, suggest that maternal size plays a significant role in shaping the fitness of SHB offspring relative to that of males.

Our study further revealed that the number of offspring produced, including larvae and adult SHBs, is directly proportional to the duration of SHB development, contrary to the standard offspring‐number versus development‐time trade‐off (Church et al. 2021; Sakai and Harada 2004). This is likely a correlation driven by variation in body size. It would be reasonable to assume that the more offspring produced in SHBs, the less maternal provision each receives, resulting in lower offspring quality and longer development. This may explain why larvae produced in large numbers took longer to develop into adults in our study. It could also help explain why adults produced in smaller numbers developed more quickly, as observed in this study. These results corroborate previous findings (Hu et al. 2015; Khokhlova et al. 2014). For instance, in cereal aphids, including *Sitobion avenae* and *Schizaphis graminum*, fecundity correlates with the development period (Hu et al. 2015). Likewise, in the flea species *Xenopsylla ramesis*, offspring produced in greater numbers develop more slowly (Khokhlova et al. 2014). In these specific examples, offspring from larger populations were relatively small, confirming our findings of a negative correlation between offspring number and weight and size. These findings indicate that variation in body size may have ecological and evolutionary implications in SHBs, given their invasive nature.

The contrasting reproductive strategies associated with SHB body size help explain why this species is such a successful and persistent invader. Big‐sized adults may produce high numbers of offspring, enabling rapid population growth when resources are abundant, whereas small‐sized adults produce fewer but higher‐fitness offspring capable of surviving under more restrictive or stressful conditions. This dual strategy likely enables SHB populations to persist across a wide range of colony sizes, resource levels, and environmental conditions. From a management standpoint, this flexibility makes control efforts particularly challenging: suppressing one phenotype does not eliminate the other, and environmental or colony‐level stressors that weaken large, highly fecund adults may simultaneously favor the survival of smaller ones. Consequently, SHB populations can rebound quickly and maintain persistence even under unfavorable conditions. Understanding this trade‐off is therefore essential for developing management strategies that disrupt both high‐output and high‐fitness pathways, rather than inadvertently selecting for the more resilient components of the population.

Differential responses to heat‐ and cold‐stress in SHB offspring were observed in this study. This is expected because temperature plays a key role in the biology and development of SHBs (Bernier et al. 2014; de Guzman and Frake 2007; Meikle and Patt 2011; Noor‐Ul‐ane and Jung 2021). Previous studies found that temperature influences the survival, reproduction, and life‐history traits of SHBs, with beetles performing better at an optimal temperature of 30°C–35°C than at 24°C–28°C (de Guzman and Frake 2007; Meikle and Patt 2011). This explains why the beetle's survival rate was significantly reduced at temperatures outside this optimal range in our study. Our results indicate that big‐sized females are more resilient to thermal stress, while small‐sized males are more vulnerable. de Guzman and Frake (2007) demonstrated that temperature shapes adult SHB size and weight, depending on larval exposure temperatures. It implies that adult body size may play a role in the thermal resilience of this invasive beetle. Big‐sized SHB females handle thermal stress more effectively because they may have lower mass‐specific metabolic rates, which helps them accumulate metabolic damage more slowly compared to smaller individuals whose tissues are closer to their metabolic limits. It will be interesting to test this hypothesis in future studies, given that smaller individuals may gain or lose heat more rapidly because of their smaller body size. Additionally, big‐sized SHB females may accumulate more lipids, glycogen, and water, helping them sustain protective responses during extended thermal stress and thereby delaying exhaustion compared to smaller individuals, who deplete their reserves quickly. This assertion may also need further study to be proven. The differential responses of SHBs of varying sizes and sexes to thermal stress observed in our study support previous findings in other insect species (Maebe et al. 2021; Nervo et al. 2021; Tonione et al. 2020; Walzer et al. 2025). However, how parental body size influences offspring fecundity or egg viability under extreme temperature regimes, which this study did not investigate, remains unclear. This warrants investigation in future studies, as previous research has demonstrated the impact of temperature on these important SHB life traits (Bernier et al. 2014; de Guzman and Frake 2007; Meikle and Patt 2011).

The fact that the beetles survived for only about 2 days at the coldest treatment temperature (5°C) in our study is intriguing, especially given that adult honey bees can survive several hours at 0°C and recover after extended periods in chill coma at near‐freezing temperatures, even though honey bee colonies can survive negative temperatures (Abramson et al. 1997; Feliciano‐Cardona et al. 2020; Sánchez‐Echeverría et al. 2019). It raises the question of whether this physiological difference could be exploited in SHB management. Of course, such an approach would require careful evaluation to avoid harming the colony or disrupting brood development, but the concept is worth exploring.

Differences in reproduction potential, offspring phenotype, and thermal tolerance that depend on body size in SHBs indicate this invasive pest has multiple, complementary life‐history strategies. These strategies can boost its ecological impact and ensure its long‐term survival in and around honey bee colonies. Big‐sized females have a demographic and climatic edge by producing more, high‐emergence offspring and demonstrating greater resilience to thermal stress, traits that support population growth and stability amid fluctuating hive and soil temperatures in their new environments. Conversely, smaller females produce fewer but larger, faster‐developing offspring, which may enhance their competitive advantage within the hive and enable better utilization of temporary brood and pollen resources. This dual strategy underlines SHB's ability to damage colonies even when development times are limited. The clear link between parental size and offspring fitness suggests that body size and related characteristics can respond quickly to selection pressures, aiding evolutionary adaptation to local climates and host environments, and explaining why SHB infestations can become severe worldwide. The fact that only the F2 generation of SHBs was examined in this study makes it worthwhile for further studies to assess long‐term transgenerational effects on body size and to validate these findings in the field. Management strategies that exploit these life‐history trade‐offs, such as prioritizing the reduction of larval nutrition and hive waste (spilled honey, pollen, and brood debris) to limit the production of large adults, while simultaneously engineering soil habitat around colonies (using dry and hard surfaces) to suppress successful development of pupae into large, thermal stress‐tolerant SHB adult phenotypes, reducing overall demographic performance and pest pressure, could be utilized to direct populations toward vulnerable generations, thereby enhancing long‐term management efficacy of this invasive pest.

## Author Contributions

**Bashiru Adams:** conceptualization (equal), formal analysis (equal), investigation (equal), validation (equal), visualization (equal), writing – original draft (lead), writing – review and editing (equal). **Jian Chen:** conceptualization (equal), formal analysis (equal), funding acquisition (equal), investigation (equal), methodology (equal), resources (equal), supervision (equal), validation (equal), visualization (equal), writing – review and editing (equal). **Esmaeil Amiri:** conceptualization (equal), funding acquisition (equal), investigation (equal), methodology (equal), resources (equal), supervision (equal), validation (equal), visualization (equal), writing – review and editing (equal).

## Funding

This research was supported by the United States Department of Agriculture, Agricultural Research Service through Cooperative Agreements #58‐6066‐9‐045 and #58‐6066‐4‐014.

## Disclosure

Our study includes authors from various countries, including those based in the study's location. All authors participated early in the research and design process to incorporate their diverse perspectives from the beginning. When applicable, we cited regional scientific literature and aimed to include relevant work published in the local language.

## Conflicts of Interest

The authors declare no conflicts of interest.

## Data Availability

The data supporting the findings of this study are openly available in the Dryad repository at https://doi.org/10.5061/dryad.w6m905r3c. All R scripts and code used for data analysis are available in the same repository.
